# Lack of association of polymorphisms in homocysteine metabolism genes with pseudoexfoliation syndrome and glaucoma

**Published:** 2008-12-26

**Authors:** Bao Jian Fan, Teresa Chen, Cynthia Grosskreutz, Louis Pasquale, Douglas Rhee, Elizabeth DelBono, Jonathan L. Haines, Janey L. Wiggs

**Affiliations:** 1 Department of Ophthalmology, Harvard Medical School, Massachusetts Eye and Ear Infirmary, Boston, MA; 2 Center for Human Genetics Research, Vanderbilt University Medical School, Nashville TN

## Abstract

**Purpose:**

To evaluate genes involved in homocysteine metabolism as secondary risk factors for pseudoexfoliation syndrome (PXFS) and the associated glaucoma (PXFG).

**Methods:**

One hundred eighty-six unrelated patients with PXFS, including 140 patients with PXFG and 127 unrelated control subjects were recruited from the Massachusetts Eye and Ear Infirmary. All the patients and controls were Caucasian of European ancestry. Seventeen tag SNPs from 5 genes (methylenetetrahydrofolate reductase [*MTHFR*], methionine synthase [*MTR*], methionine synthase reductase [*MTRR*], methylenetetrahydrofolate dehydrogenase [*MTHFD1*], and cystathionine β-synthase [*CBS*]) were genotyped. Single-SNP association was analyzed using Fisher’s exact test (unconditional) or logistic regression after conditioning on the effects of age and three *LOXL1* SNPs (rs1048661, rs3825942, and rs2165241). Interaction analysis was performed between the homocysteine and *LOXL1* SNPs using logistic regression. Haplotype analysis and the set-based test were used to test for association of individual genes. Multiple comparisons were corrected using the Bonferroni method.

**Results:**

One SNP (rs8006686) in *MTHFD1* showed a nominally significant association with PXFG (p=0.015, OR=2.23). None of the seventeen SNPs tested were significantly associated with PXFS or PXFG after correcting for multiple comparisons (Bonferroni corrected p>0.25). After controlling for the effects of age and three associated *LOXL1* SNPs, none of the seventeen tested SNPs were associated with PXFS (p>0.12). No significant interaction effects on PXFS were identified between the homocysteine and *LOXL1* SNPs (p>0.06). Haplotype analysis and the set-based test did not find significant association of individual genes with PXFS (p>0.23 and 0.20, respectively).

**Conclusions:**

Five genes that are critical components of the homocysteine metabolism pathway were evaluated as secondary factors for PXFS and PXFG. Our results suggest that these genes are not significant risk factors for the development of these conditions.

## Introduction

Pseudoexfoliation syndrome (PXFS) is a common condition characterized by the deposition of microfibrillar material throughout the eye. The composition of the PXFS-related material, although not completely defined, appears to be a complex glycoprotein structure containing elements of basement membranes and the elastic fiber system [1]. The biologic processes that cause this material to accumulate in ocular structures are not known. In eyes with PXFS, fibrillar material is found throughout the anterior segment and is typically evident on the lens capsule and in the angle where it may impede the flow of aqueous humor through the trabecular outflow pathways [2]. Over 50% of individuals with PXFS develop high-pressure glaucoma (PXFG) which may be associated with rapidly progressive optic nerve degeneration [3].

Pseudoexfoliation syndrome and the associated glaucoma appear to be genetically complex. Twin studies and reports of familial aggregation demonstrated significant heritability but not a clearly defined inheritance pattern, suggesting complex or multifactorial inheritance [4-6]. A genome-wide scan using a large Finnish family indicated potential linkage to multiple chromosome regions including 18q, 2q, 17q, and 19q [7]. Recently, a genome-wide association study identified significant association of three SNPs (rs1048661, rs3825942, and rs2165241) in the lysyl oxidase-like 1 (*LOXL1*) gene with PXFS and PXFG in patients from Iceland and Sweden [8]. This association has been replicated in our study of a USA clinic-based population with broad ethnic diversity [9] and in other studies using ethnic populations of Caucasian [10-16], Indian [17], and Japanese [18-22]. These results demonstrate that *LOXL1* is a major gene associated with PXFS and PXFG.

Two of the highly associated *LOXL1* SNPs are missense changes in exon 1 (rs3825942, G153D and rs1048661, R141L), however, it is not yet known if these variants are biologically causative or are in linkage disequilibrium with other gene variants that are biologically active. The G153D risk allele (G) frequency is very high in PXFG patients in most of the populations studied (92%–99%), but is also prevalent in control samples, with a frequency of over 65% in many populations [8-22]. In addition, in the Australian population the frequency of the rs3825942 risk allele is much higher than the disease prevalence, indicating a reduction in penetrance compared to the USA and European populations [14]. Collectively, these results suggest that additional genetic and/or environmental factors that are potentially additive and/or protective could influence the development of this complex disorder [23].

Previous reports have indicated that homocysteine is moderately elevated in aqueous humor, tear fluid, and serum plasma of patients with PXFS and PXFG [24-26]. It has been proposed that mild elevations of homocysteine may contribute to the increased vascular risk that has been observed in patients with PXFS, which includes aneurysms of the abdominal aorta [27]. It is well recognized that hyperhomocysteinemia is associated with vascular abnormalities [28], and the modest elevations reported in PXFS patients could initiate vascular damage that could be further compromised by abnormal LOXL1 activity. Variants in genes that regulate the homocysteine pathways could be responsible for the observed elevations of homocysteine and these may be additive genetic factors that influence the development of the syndrome. The purpose of this study was to evaluate 5 genes encoding enzymes that regulate homocysteine metabolism as secondary factors that could contribute to PXFS and PXFG.

## Methods

### Patients and control subjects

One hundred eighty-six patients with PXFS were recruited from the Glaucoma Consultation Service at the Massachusetts Eye and Ear Infirmary, Boston, MA. Patients with PXFS were identified by the presence of the characteristic fibrillar material on the lens capsule or pupillary margin. Patients with iris transillumination defects without the presence of the fibrillar material were not identified as pseudoexfoliation patients, or controls. Of the 186 patients with PXFS, 140 had glaucoma (PXFG) and 46 did not (PXFNG). Glaucoma was defined as: intraocular pressure >22 mmHg in both eyes on two occasions or intraocular pressure >19 mmHg in both eyes on treatment with two or more glaucoma medications; evidence of optic nerve damage in both eyes; and visual field defects consistent with optic nerve damage and characteristic for glaucoma in at least one eye. One hundred twenty-seven control subjects were recruited from the Comprehensive Ophthalmology Service at the Massachusetts Eye and Ear Infirmary, Boston, MA. Control subjects had no evidence of pseudoexfoliation or glaucoma after clinical exam. The average age of the PXFS patients was 75. Because of the age-dependence of the pseudoexfoliation syndrome, only controls older than age 60 were used for this analysis with an average age of 72. This study population (cases and controls) included only Caucasian participants of European ancestry. Fifty-nine percent of the patients were female with 41% male, while 51% of the controls were female and 49% were male. This study adhered to the tenets of the Declaration of Helsinki and has been reviewed and approved by the Institutional Review Board of the Massachusetts Eye and Ear Infirmary. Informed consent was obtained from all patients and controls.

### Gene polymorphisms and genotyping

**Figure 1:**
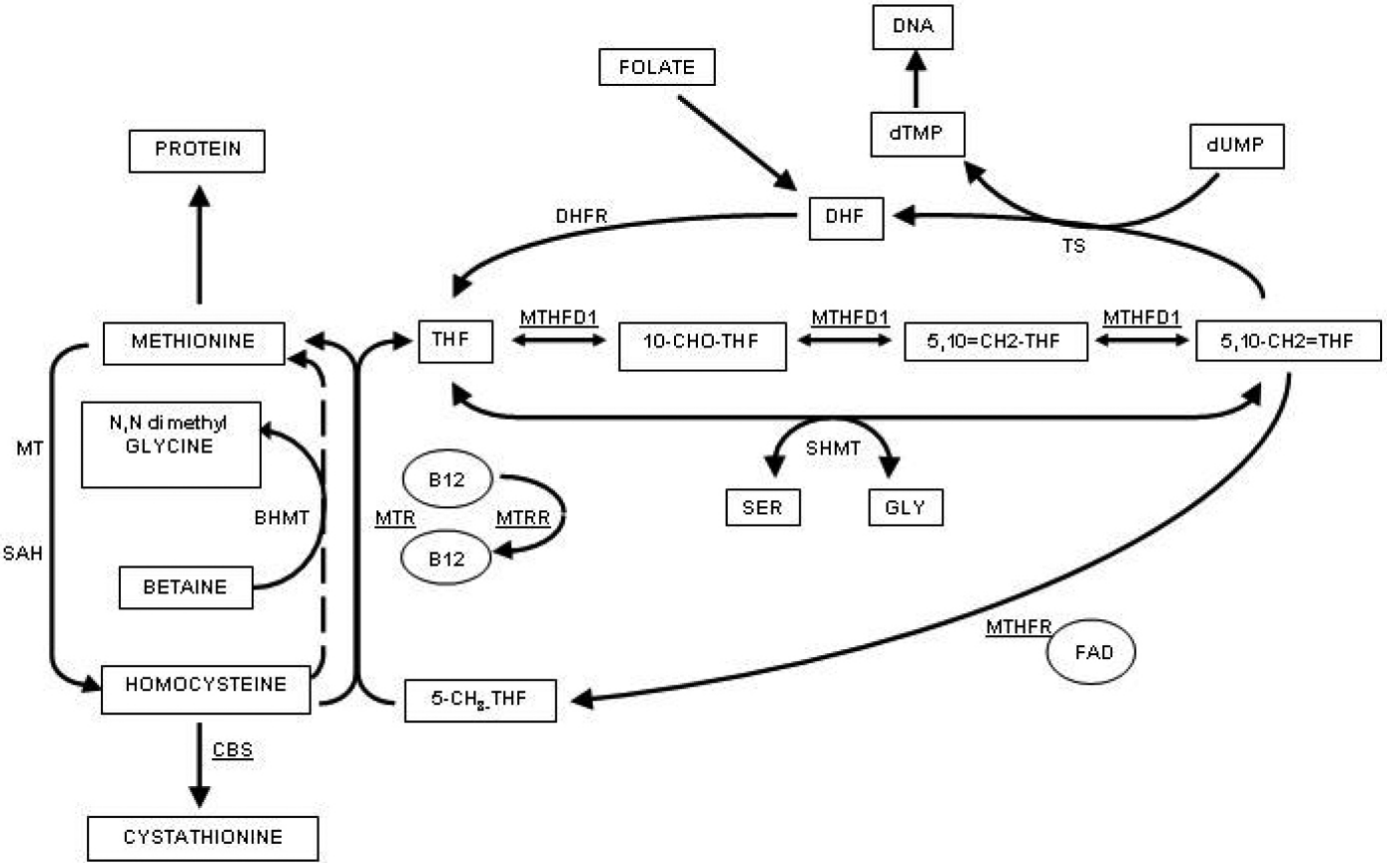
Homocysteine metabolic pathways. Products of the enzymatic pathways are shown in rectangles, co-factors are shown in circles, and enzymes are in text. The genes coding for the enzymes included in this study are shown as underlined text. Abbreviations: **B12**, vitamin B12; **BHMT,** betaine-homocysteine methyltransferase; **CBS**, cystathionine beta synthase; **DHF**, dihydrofolate; **DHFR**, dihydrofolate reductase; **dTMP**, thymidine monophosphate; **dUMP**, uridine monophosphate; **FAD**, flavin adenine dinucleotide; **GLY**, glycine; **MTHFD1**, trifunctional methylenetetrahydrofolate dehydrogenase, cyclohydrolase, synthase; **MTHFR**, methylenetetrahydrofolate reductase; **MTR**, 5-methyltetrahydrofolate-homocysteine methyltransferase; **MTRR**, 5-methyltetrahydrofolate-homocysteine methyltransferase reductase; **MT**, methyl transferase; **SAH**, S-adenosylhomocysteine hydrolase; **SER**, serine; **THF**, tetrahydrofolate; **TS**, thymidylate synthase; **5-CH3-THF**, 5-methyl tetrahydrofolate; **5,10-CH2=THF**, methylene tetrahydrofolate; **5,10=CH2-THF**, methenyl tetrahydrofolate; **10-CHO-THF**, 10-formyl tetrahydrofolate.

Five genes that encode proteins that are involved in homocysteine metabolism were investigated in this study (Figure 1). These genes are *MTHFR* (methylenetetrahydrofolate reductase), *MTR* (methionine synthase), *MTRR* (methionine synthase reductase), *MTHFD1* (methylenetetrahydrofolate dehydrogenase), and *CBS* (cystathionine β-synthase). Tag SNPs corresponding to linkage disequilibrium (LD) blocks were selected using Haploview (version 4.1) [29] according to the HapMap data (release 23a) from the CEU population. The minimum minor allele frequency for checking markers was set to 0.01. Three or 4 tag SNPs were selected for each gene to capture the majority of alleles at r^2^ greater than 0.8 across the whole gene including the 5′UTR and 3′UTR (Table 1). Each LD block was captured by 1 or 2 SNPs although not all alleles in each gene were captured. Genotyping was performed either by TaqMan assays (Applied Biosystems [ABI], Foster City, CA) or by direct sequencing. For the TaqMan assays, oligonucleotide primers were ordered from ABI (assay by demand) and performed according to the manufacturer’s instructions. For direct sequencing, products from PCR amplification were purified and sequenced using BigDye^®^ chemistries (ABI) and an automated genetic analyzer (model 3100; ABI). Sequence data was analyzed using Vector NTI suite (version 8).

**Table 1:** Characteristics and genotype counts of the 17 SNPs in 5 homocysteine genes.

**Gene**	**SNP**	**Chr**	**Position (bp)**	**Codon change**	Genotype count (AA/AB/BB)*			
					**PXFS**	**PXFG**	**PXFNG**	**Controls**
*MTHFR*	rs1801131	1	11777063	E429A	13/29/37	11/20/26	2/9/11	9/19/22
*MTHFR*	rs1801133	1	11778965	A222V	10/41/33	7/31/23	3/10/10	7/22/21
*MTHFR*	rs17037396	1	11784634	intron	0/15/71	0/13/46	0/2/25	0/17/73
*MTR*	rs12096955	1	235055726	intron	14/40/31	10/28/21	4/12/10	12/39/40
*MTR*	rs2229274	1	235056807	D294N	0/3/80	0/2/59	0/1/21	0/4/53
*MTR*	rs1805087	1	235115123	D919G	6/57/121	6/44/88	0/13/33	6/38/74
*MTRR*	rs326122	5	7929611	intron	2/29/53	1/20/37	1/9/16	5/22/62
*MTRR*	rs1532268	5	7931179	S202L	7/37/37	6/26/26	1/11/11	11/25/18
*MTRR*	rs161870	5	7931192	L206L	17/27/20	11/22/14	6/5/6	10/19/11
*MTRR*	rs162036	5	7938959	R377K	4/20/63	4/13/43	0/7/20	3/20/68
*MTHFD1*	rs8006686	14	63938424	intron	5/27/55	2/23/35	3/4/20	0/21/70
*MTHFD1*	rs2236225	14	63978598	R653Q	34/96/51	24/71/42	10/25/9	30/52/35
*MTHFD1*	rs11627387	14	63993730	intron	7/31/45	4/23/30	3/8/15	8/32/50
*CBS*	rs2124459	21	43348783	intron	16/43/29	9/39/23	7/4/6	14/40/37
*CBS*	rs1801181	21	43353685	A360A	21/75/77	13/60/58	8/15/19	14/43/49
*CBS*	rs5742905	21	43356253	I278T	1/32/151	1/25/112	0/7/39	2/10/96
*CBS*	rs234715	21	43361464	intron	4/29/55	3/25/43	1/4/12	5/30/54

The asterisk indicates that “A” represents the minor allele and “B” represents the common allele.

### Statistical analysis

Statistical analyses were performed using PLINK (version 1.04) [30]. Hardy–Weinberg equilibrium was assessed by the χ^2^ test. Linkage disequilibrium was measured using r^2^. Initial single-SNP association analysis was performed using the Fisher's exact test. Multivariable analysis of individual SNPs was performed using logistic regression models. The additive effects model was applied to analysis of allele dosage in which the genotypes AA, AB, BB were coded as 0, 1, 2, respectively, where A represents the minor allele and B represents the common allele. After controlling for the effects of age and three significant *LOXL1* SNPs (rs1048661, rs3825942, and rs2165241), individual SNPs in the homocysteine genes were analyzed for association with PXFS using logistic regression. Interaction analysis of pair-wise SNPs between the homocysteine SNPs and the *LOXL1* SNPs was performed by including an interaction term in the logistic regression models. Haplotype analysis and the set-based test were used to test for association of individual genes. Haplotype frequencies were estimated using the standard E-M algorithm and tested using the χ^2^ test. The omnibus p-values for haplotype analysis were obtained from the omnibus test. The set-based test selects the best set of SNPs whose mean of these single SNP statistics is significant after permutation, which is particularly suited to large-scale candidate gene studies [31]. The empirical p values of the set-based test were obtained by a permutation of 10,000 times of phenotype labels. Multiple comparisons were corrected using the Bonferroni method.

## Results

### Single-SNP association analysis

Seventeen SNPs in five genes were analyzed for association with PXFS, PXFG, and PXFNG (Table 1). All of these SNPs followed Hardy–Weinberg equilibrium in both cases and controls (p>0.05). The selected SNPs were not in strong LD (r^2^<0.26), which is consistent with the HapMap data from the CEU population. Only one SNP, rs8006686 in *MTHFD1*, showed a marginally significant association with PXFS (p=0.015; OR=2.07, 95%CI: 1.16, 3.71) and PXFG (p=0.015; OR=2.23, 95%CI: 1.19, 4.16). However, none of these SNPs were significantly associated with PXFS, PXFG or PXFNG after correcting for multiple comparisons (Bonferroni corrected p>0.25; Table 2).

**Table 2:** Single-SNP association of homocysteine genes with PXFS, PXFG and PXFNG.

**Gene**	**SNP**	**Minor allele**	**Minor allele frequency**				p value*		
			**PXFS**	**PXFG**	**PXFNG**	**Controls**	**PXFS**	**PXFG**	**PXFNG**
*MTHFR*	rs1801131	C	0.348	0.368	0.295	0.370	0.79	1.00	0.45
*MTHFR*	rs1801133	T	0.363	0.369	0.348	0.360	1.00	1.00	1.00
*MTHFR*	rs17037396	T	0.087	0.110	0.037	0.094	0.85	0.70	0.26
*MTR*	rs12096955	T	0.400	0.407	0.385	0.346	0.32	0.33	0.62
*MTR*	rs2229274	A	0.018	0.016	0.023	0.035	0.45	0.43	1.00
*MTR*	rs1805087	G	0.188	0.203	0.141	0.212	0.47	0.83	0.16
*MTRR*	rs326122	G	0.196	0.190	0.212	0.180	0.78	0.88	0.69
*MTRR*	rs1532268	T	0.315	0.328	0.283	0.435	0.053	0.10	0.10
*MTRR*	rs161870	A	0.477	0.468	0.500	0.488	0.89	0.88	1.00
*MTRR*	rs162036	A	0.161	0.175	0.130	0.143	0.66	0.52	1.00
*MTHFD1*	rs8006686	C	0.213	0.225	0.185	0.115	0.015	0.015	0.25
*MTHFD1*	rs2236225	T	0.453	0.434	0.489	0.479	0.56	0.33	0.62
*MTHFD1*	rs11627387	G	0.271	0.272	0.269	0.267	1.00	1.00	1.00
*CBS*	rs2124459	C	0.426	0.401	0.471	0.374	0.33	0.65	0.13
*CBS*	rs1801181	T	0.338	0.328	0.369	0.335	1.00	0.92	0.59
*CBS*	rs5742905	C	0.092	0.098	0.076	0.065	0.28	0.25	0.81
*CBS*	rs234715	G	0.210	0.218	0.176	0.225	0.80	1.00	0.65

The asterisk indicates that p values were obtained from Fisher’s exact test when compared to controls. The Bonferroni corrected significance level was 0.003 (0.05/17).

After controlling for the effects of age and the three significant *LOXL1* SNPs (rs1048661, rs3825942, and rs2165241), logistic regression analysis also showed that none of the SNPs in the homocysteine genes were associated with PXFS (p>0.12; Table 3). No significant interaction effects on PXFS were found between the homocysteine SNPs and the *LOXL1* SNPs (p>0.06; data not shown).

**Table 3:** Single-SNP association of homocysteine genes with PXFS after controlling for the effects of age and 3 *LOXL1* SNPs.

**Gene**	**SNP**	**Minor allele**	p value*	OR (95% CI)*
*MTHFR*	rs1801131	C	0.95	0.98 (0.49, 1.96)
*MTHFR*	rs1801133	T	0.55	0.79 (0.37, 1.70)
*MTHFR*	rs17037396	T	0.81	0.89 (0.33, 2.40)
*MTR*	rs12096955	T	0.23	1.43 (0.80, 2.54)
*MTR*	rs2229274	A	0.55	0.52 (0.06, 4.41)
*MTR*	rs1805087	G	0.69	0.90 (0.52, 1.55)
*MTRR*	rs326122	G	0.95	1.02 (0.50, 2.07)
*MTRR*	rs1532268	T	0.82	1.09 (0.50, 2.38)
*MTRR*	rs161870	A	0.46	0.74 (0.33, 1.65)
*MTRR*	rs162036	A	0.91	0.96 (0.49, 1.90)
*MTHFD1*	rs8006686	C	0.21	1.62 (0.76, 3.45)
*MTHFD1*	rs2236225	T	0.77	0.93 (0.59, 1.47)
*MTHFD1*	rs11627387	G	0.24	0.68 (0.36, 1.29)
*CBS*	rs2124459	C	0.71	1.13 (0.61, 2.10)
*CBS*	rs1801181	T	0.49	1.18 (0.74, 1.89)
*CBS*	rs5742905	C	0.12	2.07 (0.84, 5.14)
*CBS*	rs234715	G	0.57	0.80 (0.36, 1.74)

The asterisk indicates that p values and odds ratios (OR) with 95% confidence intervals (CI) were obtained from logistic regression analysis of single-SNP association of homocysteine genes with PXFS after controlling for the effects of age and the 3 *LOXL1* SNPs (rs1048661, rs3825942, and rs2165241).

### Gene-based association analysis

Haplotype association analysis of all the tag SNPs in each gene revealed no association of individual genes with PXFS (omnibus p>0.23; Table 4). Set-based association tests also did not identify significant association of individual genes with PXFS (empirical p>0.04, Bonferroni corrected p>0.20; Table 4).

**Table 4:** Gene-based association of homocysteine genes with PXFS.

**Gene**	**SNPs**	**p value**	
		Haplotype test*	**Set-based test^#^**
*MTHFR*	rs1801131 , rs1801133, rs17037396	0.97	1.00
*MTR*	rs12096955 , rs2229274, rs1805087	0.68	1.00
*MTRR*	rs326122 , rs1532268, rs161870, rs162036	0.85	0.19
*MTHFD1*	rs8006686 , rs2236225, rs11627387	0.30	0.04
*CBS*	rs2124459 , rs1801181, rs5742905, rs234715	0.23	1.00

The asterisk indicates that the p values were obtained from the omnibus haplotype test using PLINK (version 1.04) [30] and the the sharp (hash mark) indicates that the p values were obtained from the set-based test by a permutation of 10,000 times using PLINK (version 1.04) [30]. The Bonferroni corrected significance level was 0.01 (0.05/5).

## Discussion

Recent studies suggest that *LOXL1* is a major gene associated with PXFS/PXFG, contributing to the majority of cases in most populations [8-22]. However, the high prevalence of the rs3825942 risk allele in control populations, and the apparent variable penetrance of the condition in some populations suggest that additional genetic factors and/or environmental exposures could be involved in the development of this complex disease. As moderate hyperhomocysteinemia has been repeatedly described in PXFS and PXFG patients [24-26], we evaluated the genes that code for proteins involved in homocysteine metabolism as candidates for secondary factors contributing to this disease.

Multiple reports have indicated that patients with PXFS have mild elevations of homocysteine in serum plasma, as well as aqueous humor and tear fluid [24-26]. As the association of hyperhomocysteinemia with vascular disease has been well documented [28], and ocular and systemic blood vessels in PXFS can be abnormal [32], we hypothesized that elevated homocysteine caused by variant forms of genes coding for key enzymes involved in homocysteine metabolism could contribute to the PXFS. Defects in LOXL1 can also compromise the elastic structure of blood vessels [33], and that the combined effects of elevated serum homocysteine and LOXL1 deficiency could synergistically contribute to vascular compromise. Abnormalities of ocular vasculature, especially blood vessels in the iris could be related to the deposition of the microfibrillar material that is characteristic of the disease process. Previous studies have failed to show an association between homocysteine metabolism genes and PXFS [34-39], however these studies have evaluated only the well studied common C677T polymorphism (rs1801133) in *MTHFR*, a central regulator of homocysteine levels. In this study we took a broader approach and evaluated 5 genes involved in homocysteine metabolism, including the *MTHFR* gene. We did not find any significant association between any of the homocysteine genes and PXFS and/or PXFG in this present study.

Cystathionine β-synthase catalyzes the transsulfuration of homocysteine to cystathionine. The *CBS* c.844_845ins68 mutation has been associated with increased CBS enzyme activity and decreased homocysteine levels [40,41], and has been hypothesized to have a protective effect against vascular thromboembolic disease [42]. We initially sequenced a sample of 100 patients with PXFS and 100 controls and observed that another *CBS* variant, rs5742905 (I278T), was in complete linkage disequilibrium with c.844_845ins68 (r^2^=1.0). We therefore considered rs5742905 as a surrogate for c.844_845ins68 and only genotyped rs5742905 in our subsequent samples using TaqMan assays. In our population, we did not find any association of rs5742905 with PXFS or PXFG, and so did the c.844_845ins68 mutation.

As age and *LOXL1* variants are two known major risk factors for PXFS and PXFG, we analyzed the association between SNPs in homocysteine metabolism genes and PXFS and PXFG using logistic regression after controlling for the effects of age and the *LOXL1* variants. The *MTHFD1* SNP rs8006686 was marginally associated with PXFG before correction for multiple comparisons (p=0.015; Table 2). However, this association disappeared after controlling for the effects of age and the *LOXL1* variants (p=0.21; Table 3). To increase the statistical power to identify a possible association, we further analyzed our data using haplotype analysis and the set-based test, both of which are gene-based tests where all SNPs in a gene are analyzed together. The set-based test is particularly suited to large-scale candidate gene studies [31]. This method selects the best set of SNPs whose mean statistic is significant, leading to the inference that the entire set of SNPs might be interacting in some way to increase disease risk, or else that they are all contributing independently to disease risk. In the present study, both haplotype analysis and the set-based test did not find any significant association between the homocysteine genes and PXFS or PXFG (Table 4), in agreement with the logistic regression analysis of single SNPs in the homocysteine genes after controlling for the effects of age and the *LOXL1* variants (Table 3).

We estimated that the present study had 86% of power to detect a moderate genetic effect (genotypic relative risk of 2.0 for Aa and 4.0 for AA, given an additive risk model) [43]. However, this study had only 40% of the power needed to detect a mild genetic effect (genotypic relative risk of 1.5 for Aa and 2.25 for AA, given an additive risk model). In addition, since we used tag SNPs to capture the majority of the variants in each gene, it is possible that we might have missed other variants in these genes associated with the disease. Further large-scale studies and resequencing of the whole genes are warranted to confirm our findings.

Dietary factors that are important regulators of homocysteine metabolism have also been shown to be abnormal in PXFS and PXFG patients including low levels of B6, B12, and folate [44]. It is possible that diets that are low in these vitamins may contribute to these conditons by causing and elevation of homocysteine levels with subsequent interaction with the vascular insult caused by defective LOXL1. Although further documentation is necessary before disease risks can be determined, individuals who are carriers of the *LOXL1* at risk genotypes should be encouraged to maintain adequate levels of B6, B12, and folate in their diet.

In summary, five genes that code for critical components of the homocysteine metabolism pathway were evaluated as secondary factors for PXFS/PXFG in the present study. Our results suggest that variants in these genes are not major risk factors for the development of these conditions. Other important regulators of homocysteine metabolism, such as dietary intake of B6, B12, and folate may be contributing secondary environmental factors. Further studies searching for secondary genetic and environmental factors that contribute to PXFS and PXFG are required to gain a better understanding of the complex etiology of this important ocular disease.
